# Covert Exogenous Cross-Modality Orienting between Audition and Vision †

**DOI:** 10.3390/vision2010008

**Published:** 2018-02-09

**Authors:** Raymond M. Klein

**Affiliations:** Department of Psychology and Neuroscience, Dalhousie University, Halifax, NS B3H 4R2, Canada; ray.klein@dal.ca

**Keywords:** cross-modal attention, exogenous orienting, vision, audition

## Abstract

Control of visual attention by auditory stimuli is explored in seven previously unpublished experiments that were presented at conferences in the late 1980s. Reaction time (RT) to luminance targets was found to be affected by the spatial congruence between the target and a preceding or simultaneous, and non-informative, auditory event, suggesting that localizable auditory stimuli exogenously (rapidly and automatically) capture visual attention. These cuing effects were obtained in the absence of eye movements and do not appear to be mediated merely by criterion adjustments. When the information value of the auditory event was placed in conflict with its location (i.e., a tone on the right indicated that the visual target was likely to appear on the left), it was found that at short stimulus onset asynchronies (SOAs) reaction time (RT) was faster for targets at the stimulated location, an effect that disappeared within 500 ms and was reversed by 1000 ms. This demonstrates that it requires over 500 ms for endogenous orienting in response to probabilistic information about target location to overcome the powerful exogenous control of visual attention by localizable auditory stimulation. Simple RT to auditory stimuli was unaffected by the spatial congruence of a preceding or simultaneous visual stimulus. When uninformative, neither pitch contours (rising/falling tones) nor pitch (high/low tones) produced significant visual orienting along the vertical midline. When the direction of a pitch contour indicated the likely location of a visual target, participants were able to shift their attention if the relation between the natural meaning and the probabilistic information was compatible (e.g., rising contour signaled that a upper target was likely) but not when it was incompatible. The relation of these 30-year-old experiments to contemporary findings and ideas is discussed.

## 1. Introduction

Spatial orienting refers to adjustments that improve the processing of stimuli coming from a location in space or that improve their access to response mechanisms [1] (Posner, 1978). Posner (1980) [2] put forward two distinctions to help classify different forms of orienting (see also, Klein, 2005) [3]. When such adjustments are accomplished by mechanical changes in the sensory apparatus (e.g., shifts in gaze; pinna movements) they are referred to as overt. Covert orienting, on the other hand, is accomplished by internal “attentional” adjustments. Both covert and overt orienting can be under the exogenous or reflexive control by stimuli in the environment or under the endogenous or voluntary control of the observer.

The work described here is primarily about covert shifts of attention in visual space under the control of stimuli presented to the auditory modality. The ability of stimuli presented to modalities other than vision to attract overt orienting in the form of eye movements is widely accepted and the neural machinery responsible for making saccades to localized auditory and tactile stimuli is well established (Meredith and Stein, 1986) [4]. Although our primary concern is with the ability of auditory stimuli to exogenously generate a covert shift of visual attention, in some experiments that are designed to characterize the nature of this control, we will make the auditory stimuli informative; and in one study we will explore the converse possibility. All of the reported experiments use a variant of Posner’s cuing paradigm.

In Part I of this paper we (Klein, Brennan and Gilani, 1987 [5]; Klein, Brennan, D’Aloisio, D’Entremont and Gilani, 1987 [6]) will demonstrate covert, exogenous, cross-modality orienting, or more specifically, an attentional shift toward localizable auditory that affects the processing of localized visual stimuli. One finding in Part I was that neither the pitch of a pure tone nor the direction of a frequency glide elicited an exogenous shift of visual attention along the vertical axis. In Part II we (Klein and Juckes, 1989 [7]) replicated this finding and extended it in several ways. Notably, when the frequency glide probabilistically predicted the location of the upcoming visual target participants were able to use this information when it was compatible with the natural meaning of the glide (e.g., rising pitch; upper visual target), but not when it was incompatible. Parts I and II of this paper are based on research conducted and presented in the late 1980s. To preserve “history” (e.g., the original rationales and discussions), the material in these parts has intentionally not been updated. In Part III the relation of the work presented in Parts I and II to more contemporary findings will be discussed.

## 2. Part I: Visual Orienting toward the Source of Auditory Stimulation

The studies we were aware of that presented spatial cues to one modality and targets to another used informative pre-cues which indicated the location likely to contain the target. Posner (1978) [1], for example, describes a series of studies in which informative visual precues, arrows presented at fixation, were used to direct attention spatially within the visual, tactile and auditory modalities. Whereas the results demonstrated interesting modality differences, they are not relevant to the question of exogenous cross-modality orienting because the interval between the cue and target was rather long (one second), and the subjects were presumably responding to the information value of the informative pre-cues. Buchtel and Butter (1988) [8] compared the effects of visual and auditory pre-cues upon visual and auditory simple RT over a wide range of cue-target SOAS. Unlike Posner’s cues, which were symbolic (arrows), their cues were spatial (localizable sounds and lights). However, as with the Posner studies, Buchtel and Butter’s pre-cues were highly informative about target location. Thus, the cuing effects that Buchtel and Butter observed are due to some unknown combination of exogenous orienting directed by the stimulus and endogenous orienting directed by the meaning of the cue.

It is usually assumed that performance is improved for targets presented at an attended locations and that performance is correspondingly degraded for targets elsewhere. In principle these two effects (often referred to as benefits and costs) can be separated by using a neutral condition. In practice, however, it is often difficult to produce a neutral cue that differs from the non-neutral cues only with respect to this control over attention (e.g., Jonides and Mack, 1984 [9]). Whereas the experiments we will report below each have a neutral condition, our focus will be upon the total cuing effect (uncued RT minus cued RT), which is often referred to as costs plus benefits.

### 2.1. Experiment 1

We thought it possible, if not likely, that in the absence of eye movements, irrelevant but localizable auditory events would attract visual attention (that is, produce exogenous, covert visual orienting). The same mechanisms that tend to elicit overt shifts of attention in response to localizable stimulation might also drive internal adjustments even when overt shifts are precluded. In addition to location, we thought that the frequency composition of auditory stimulation might elicit shifts of visual attention. In audio-visual media such as film and animation, pitch and pitch changes are sometimes used in conjunction with, and as if to indicate, visual location or visual movement. A rising pitch contour, for example might accompany a rising ball or spaceship; or a falling pitch contour might accompany a character falling off a cliff. The opposite relationships would seem anomalous. Psychophysical studies of cross-modality scaling (Mudd, 1963 [10]) and intersensory interactions in both affective judgment (Cohen, 1989 [11]) and discrimination time (Bernstein and Edelstein, 1971 [12]) support the idea that the pitch domain may map onto visual space in this manner. Is this cross-modality similarity, or compatibility effect wired into the nervous system in such a way that visual attention would be modified by pitch or pitch changes? The first experiment was designed to explore both these questions; more precisely: Do localizable sounds and pitch changes produce exogenous, covert orienting in visual space?

#### 2.1.1. Methods

**Figure 1:**
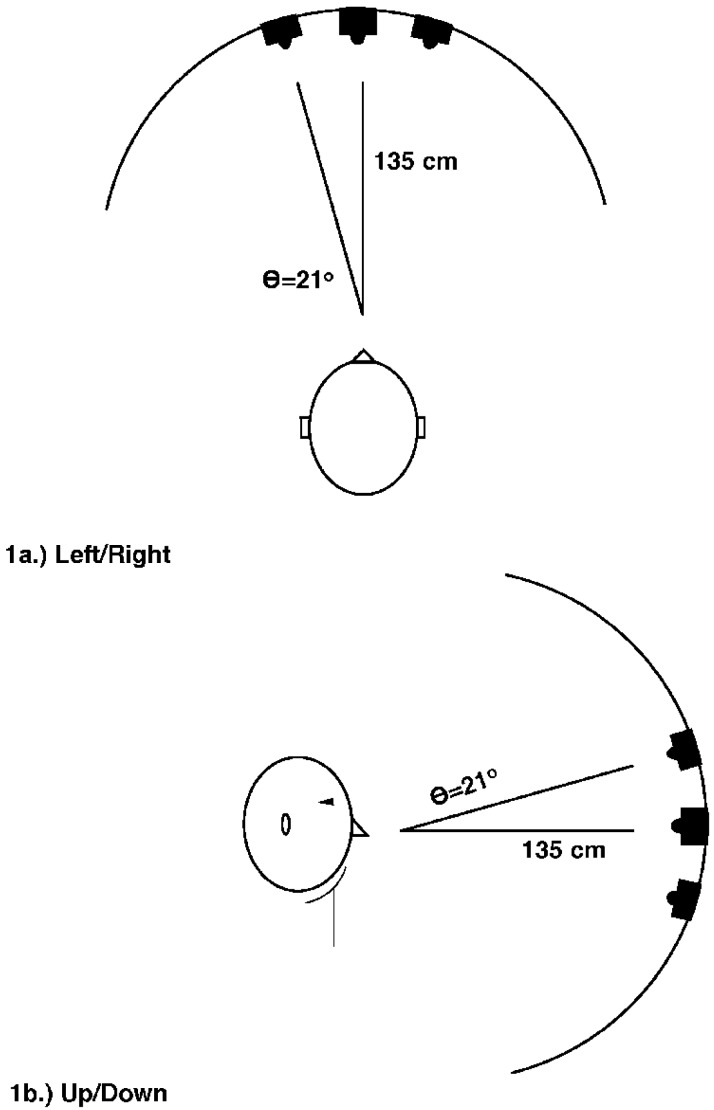
Schematic diagram showing the layout of the apparatus. (**a**) shows the layout for the left/right condition of Experiment 1 and for experiments 2–5; (**b**) shows the layout for the rising/falling condition of Experiment 1, the pure tone experiment described in [13] and Experiments 6 and 7.

Six students taking classes at Dalhousie University were paid for their participation. The apparatus and set up is illustrated in Figure 1. Red light emitting diodes (LEDs) were mounted in the middle of the front of three speakers (Realistic 40-133C), which were in turn mounted on a circular curtain rod. The subjects head was placed in a chin rest positioned 135 cm from the speakers such that the curved rod was an arc of an imaginary circle with the participant’s head at the center. At this viewing distance the LED’s subtended a visual angle of about 0.2 deg. The distance between speakers, center-to-center, was 21 deg. The rod was mounted on a stand and could be rotated to provide a horizontal (Figure 1a) or vertical (Figure 1b) arc. The subject used a hand held push button to initiate each trial and a piano style microswitch for responses to the targets.

The auditory stimuli were 250 ms in duration and were generated digitally with a “sampling” rate of 10 kHz and a constant amplitude and stored on hard disk for later use. When these stored sounds were presented (via D-A converter and speakers) they ranged in intensity from about 72 to 82 decibels (C scale) at the subject’s location. The visual stimuli consisted of the illumination of one or more LEDs, as described below.

There were two conditions, differentiated by the nature of the auditory cues and orientation of the rod, which were presented to each subject in alternating blocks. In the left-right condition the rod was horizontally oriented (Figure 1a). In this condition the auditory stimulus was a steady 800 Hz tone, which could be presented on the left, middle or right speaker. A pilot study demonstrated that the direction of the left and right auditory tones could be quickly and accurately identified. In the rising-falling condition, the rod was rotated 90° to a vertical orientation (Figure 1b). There were 3 different auditory stimuli: a steady 800 Hz tone; a rising pitch contour (700–1200 Hz) and a falling pitch contour (900–400 Hz). In this condition the tones always emanated from the central speaker.

Each trial began with the onset of the middle LED, which remained present for the duration of the trial. When the subject was fixating it and ready for the trial to begin he or she pressed the hand-held microswitch. Subjects were instructed to maintain fixation for the duration of the trial. 500 ms later the auditory cue was presented. This was followed by or accompanied by the onset of one of the peripheral LEDs. The target LED remained present until the response or for at most one second. Three different SOAS (0 ms, 250 ms and 500 ms.) were used to map out the time course of any attentional shift generated by the auditory stimuli. The subject’s task was to press the micro-switch as soon as either peripheral LED was illuminated. On 33% of the trials there was no imperative stimulus. These catch trails were used to discourage the subject from making anticipatory responses.

The probability that the left or right LED would be illuminated was unaffected by the nature of the auditory stimulus, and the subjects were carefully instructed about this uninformative relationship. Therefore there was no reason for the subjects to endogenously shift their attention in response to the cues.

Subjects were tested in 4 sessions. In each session each of the three SOAS was tested once in the left/right and once in the rising/falling condition, with SOA order balanced across subjects and sessions. Each experimental block consisted of 72 randomly ordered trials with the following breakdown: There were 16 trials with the target occurring at the cued location (e.g., left tone-left LED in the left/right condition; rising tone-top LED in the rising falling condition), 16 trials with the target occurring at the uncued location, 16 neutral trials (middle speaker in left/right condition; steady tone in rising/falling condition), and 24 catch trials (no target followed the auditory cue; with 8 neutral tone cues and 16 non-neutral tone cues). In the first session, the first experimental block in each condition was preceded by a 18 trial practice block modeled on the experimental block.

#### 2.1.2. Results and Discussion

The results are shown in Table 1. Statistical analysis of the results from the left-right condition revealed a significant effect of cue condition (F (2, 10) = 16.19, *p* < 0.001), with faster responses to targets on the cued side than the uncued side, and with the neutral cue (middle tone) condition in between. Visual inspection and statistical analysis of the data from the rising/falling condition reveals that pitch contours did not produce a significant attentional shift (F < 1).

These results demonstrate that localizable auditory events can cause an exogenous shift of visual attention and that this effect seems to be fully blown at an SOA of 0 ms. On the other hand, the rising and falling pitch contours we used did not produce significant covert orienting within the 500 ms period tested. We have since generalized this null result to high and low pure tones [13]. Although null results are often not very meaningful, in this case it should be noted that the null result with the pitch contours in Experiment 1 was accompanied by a significant attentional effect with the localizable tones. In this context, many uninteresting explanations for the null effect (such as insufficient power, uncooperative subjects) are mitigated, and the null finding gains some credibility.

### 2.2. Experiment 2

Are the cuing effects we have observed in the left/right condition produced by overt visual orienting? Since we did not monitor eye position in Experiment 1, it might be claimed that our effects are due to rapid shifts of fixation in response to the auditory cues. That the effect is observed at 0 ms SOA is not consistent with this possibility. Nevertheless, it seemed appropriate to determine more directly if we have observed overt or covert visual orienting in response to the localizable tones by replicating the left/right condition of Experiment 1 while monitoring horizontal eye position.

#### 2.2.1. Method

As the method for this experiment was that similar to that for Experiment 1, only the differences will be noted. Seven subjects participated in two sessions for payment or course credit. Only the left/right condition was tested and in each session there were two experimental blocks using each SOA. Horizontal eye position was monitored using an infra-red corneal reflection device (Eye Trac 210). Each block of trials was preceded by a calibration procedure. As before, the subject initiated the trial when he/she was fixating the central LED. At this moment the subject’s horizontal eye position was recorded. If a shift in eye position of two degrees or more was detected at any time during the trial it was aborted (no target was presented) and the subject was given feedback in the form of the illumination of all 3 LEDs and a 100 Hz tone for 250 ms. Trials that were so aborted were tabulated and later replaced.

#### 2.2.2. Results and Discussion

The results for this experiment are shown in Table 2. Statistical analysis revealed a significant effect of cue condition (F (2, 12) = 16.41, *p* < 0.001). Although the interaction between cue condition and CTOA was not significant (F < 1) the trend of smaller cuing effects at the shortest CTOA is notable. To improve power for detecting a cuing by CTOA interaction a combined analysis was performed on the data from this experiment and the left/right data from Experiment 1. In that analysis, there was a significant effect of cue condition (F (2, 22) = 32.18, *p* < 0.001) that did not interact with any variables. The effect of CTOA was also significant (F (2, 22) = 4.71, *p* < 0.25), with the 250 ms SOA producing slightly faster responses (313) than the 0 and 500 ms SOA conditions (325 and 328 respectively). When the combined data from the 0 ms SOA condition was subjected to a separate analysis the effect of cue condition was significant (F (2, 24) = 5.79, *p* < 0.01).

It is clear from these results that the cuing effects we have so far observed with localizable (left/right) auditory stimuli are due to covert rather than overt visual orienting. In all subsequent experiments, horizontal or vertical eye position was monitored, and trials with eye movements were excluded from data analysis and replaced.

It might seem surprising that visual orienting in response to auditory cues is so rapid, indeed more rapid than in response to visual cues (Buchtel and Butter, 1988) [8]. To quell any concerns arising from this surprise, we note, firstly, that a full blown cuing effect at a 0 ms SOA does not necessarily mean that visual attention is drawn to the cued location instantly. An indeterminate amount of visual processing may take place before the effects of orienting toward the visual pathway of the target are manifest in simple reactions that take 300 or more milliseconds to initiate. Moreover, there are several findings that are quite consistent with our finding of rapid covert visual orienting to auditory stimuli [14]. In one previous study (Klein, 1977) [15] auditory localization was about 80 ms faster than visual localization. Since uninformative peripheral visual events produce asymptotic cuing effects within about 100 ms it is not surprising that auditory tones can produce asymptotic cuing effects with a 0 ms SOA. More direct evidence comes from a study (Whittington, Hepp-Reymond and Flood, 1981) [16] in which monkeys were trained to fixate auditory events. After training, they could fixate the auditory stimuli more rapidly than the visual training stimuli. This finding, which demonstrates that “overt” visual orienting may be more rapid in response to auditory than to visual events also suggests that “covert” visual orienting could show the same relationship.

### 2.3. Experiment 3

We have so far demonstrated that covert visual orienting can be elicited by localizable tones. Although we believe that this covert orienting is exogenous and automatic, it is still reasonable to ask what role subject’s strategies (endogenous orienting) may play in the observed pattern of results. Recall that our cues have been uninformative about the target location, and thus following all cues ideal observers should try to attend the two possible locations equally. Nevertheless, it is possible that subjects are not ideal observers and voluntarily direct their attention toward the cue. The next two experiments put endogenous and exogenous mechanisms in conflict by having a LEFT tone mean that the visual target is likely to occur on the RIGHT and vice versa. If the orienting we have demonstrated is completely strategic, then it should be reversed by these contingencies. However, if the auditory events automatically “pull” attention to their perceived location, then it should take some time for the subjects to shift their attention to the opposite location, which is likely to contain the target. We initially thought that 500 ms would be sufficient for subjects to overcome the proposed reflexive allocation of attention to the cue, and re-orient attention toward the location likely to contain the target. Thus, Experiment 3a used SOAs of 0, 250 and 500 ms. As indicated below, the cuing effect did decrease over 500 ms, but did not reverse. Therefore, we ran Experiment 3b with SOAs of 0, 500 and 1000 ms.

#### 2.3.1. Methods

In Experiment 3a, nine subjects were tested in two sessions, with each SOA (0, 250 and 500 ms) presented twice in each session. Unlike the preceding experiments, the left and right auditory events were informative. Following either of these stimuli, the target was 3 times more likely to be presented at the uncued than at the cued location (in the terminology of endogenous orienting studies, cue validity was 75%). Subjects were not informed of the precise probabilities, but were told that the visual targets were usually presented on the speaker opposite from the auditory cues. Experiment 3b was identical to Experiment 3a with the following exceptions: 6 subjects were tested, the SOAs were 0, 500 and 1000 ms, and subjects were given the explicit information about cue validity: “that a left or right tone indicated that if there was a visual target 75% of the time it would occur on the side opposite the tone”.

#### 2.3.2. Results and Discussion

**Figure 2:**
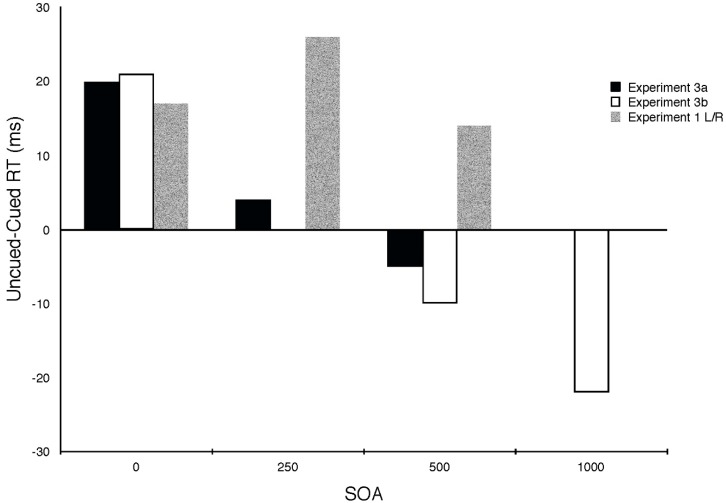
Cuing effects (uncued RT minus Cued RT, where cued refers to trials when the target appears at the location of the auditory cue) from Experiments 3a and 3b (filled bars) and, for comparison purposes, from the left/right condition of Experiment 1 (unfilled bars) as a function of SOA.

The results of Experiments 3 are shown in Table 3 and Figure 2. In both experiments there were significant interactions between SOA and cue condition (F (4,42) = 3.02, *p* < 0.05 and F (4,20) = 8.9, *p* < 0.001, for E3a and E3b respectively). There was a trend for RT to increase with SOA, but this was only significant in E3a (F (2, 16) = 10.06, *p* < 0.005). The following points can be easily seen by examining the cuing effects plotted in Figure 2: (1) attention is oriented toward the cued location (the term “cued” will be used to refer to the location of the tone) at the earliest SOA (0 ms); (2) this cuing effect was similar in magnitude to that observed in Experiment l; (3) the advantage for the cued location disappeared within 500 ms (in both experiments) and (4) one second after the auditory cue, attention was oriented at the uncued (but likely to be targeted) location.

Given the non-explicit nature of the instructions for Experiment 3a, it was possible that re-orienting was not completed by 500 ms because subjects were not well aware of the contingencies. This is an unlikely explanation given that subjects participated in 6 blocks (plus practice) in which there was a 3:1 ratio favoring the uncued location. Moreover, the fact that the data for the 500 ms SOA condition in the two experiments was very similar shows that subjects did not need explicit instructions to be fully aware of the contingencies. Together, the results of the two studies strongly support the idea that the initial response to the left/right tones is a rapid, exogenously controlled shift of visual attention to the cued location. It also appears to take between 500 ms and one second for endogenous orienting in response to the information value of the tone to overcome the exogenously generated shift in response to the tone’s location in space. We inferred that in the absence of any information value of the left/right tones (as in Experiments 1 and 2), subjects merely leave attention at the cued location after it has been pulled there by the cue.

### 2.4. Experiment 4

Are subjects in our studies obtaining RT benefits and costs by reducing their criterion (the amount of evidence required to initiate their response) for targets on the cued side, and/or correspondingly raising it on the uncued side? Evidence from several domains (e.g., Posner, Snyder and Davidson, 1980 [17]; Mangun, Hansen and Hillyard, 1986 [18]) converges on the conclusion that the effects of spatial pre-cues upon performance are not likely produced by a flexible adjustment of decisional criteria. Arguments by Sperling and others (Sperling, 1984 [19]; Duncan, 1981 [20]; Shaw, 1980 [21]) and recent data (e.g., Muller and Findlay, 1987 [22]), however, provide fuel for the criterion-shift point of view. Importantly, it is not possible, purely on the basis of the simple RT data we have presented here, to determine whether the cuing effects we have demonstrated are due to genuine changes in the efficiency of information processing (e.g., sensitivity changes) or to lowered criteria for responding to information accumulating at the attended locus. One way to assess this possibility is to conduct a choice RT task in which participants must judge a property of the target that is orthogonal to the cuing dimension. A lower, or more risky criterion will result in faster but less accurate performance, while a higher, more conservative criterion will result in slower and more accurate performance. In this experiment a speeded, 2-alternative forced-choice task was combined with left/right uninformative auditory precues to provide information relevant to this issue.

#### 2.4.1. Methods

Five subjects were tested in a single session using an SOA of 250 ms. After the practice block, there were 5 blocks of 72 experimental trials consisting of 24 cued, 24 uncued and 24 neutral trials. The targets consisted of the left or right LED flashing on for 50 or 100 ms. A 2-choice visual discrimination task was used in which subjects had to decide if the LED that flashed was short or long (or dim vs. bright—the brightness difference, due to time/intensity reciprocity was phenomenologically more salient than the duration difference).

#### 2.4.2. Results and Discussion

The results are shown in Table 4. We found a significant (F (2,8) = 23.71, *p* < 0.001) 46 ms, cuing effect in reaction time in the absence of any difference in accuracy (F < 1). If subjects had achieved their faster RTs for stimuli at the cued location by lowering criteria for initiating response to information at the cued location, the RT effect should have been accompanied by a reverse error trend. Since such a trend was not observed, it seems unlikely that the cuing effects we have obtained are due to criterion shifts.

### 2.5. Experiment 5

We thought it would be interesting to see if peripheral visual events would produce orienting in auditory space. If they did, then simple auditory RT might be affected by whether a prior or simultaneous visual cue was at the same or different location as the tone. To test this possibility, the roles of the visual and auditory stimuli were reversed. In a similar experiment, but one using centrally presented arrow cues which informed the subject of the likely location of the auditory stimuli, Posner (1978) [1] found that the informative pre-cues did not speed the detection or discrimination of auditory stimuli. By exploring the question in the context of exogenous orienting, the present study may also help provide evidence concerning the reason for Posner’s null result. For example, if it were due to an insensitivity of auditory stimuli to the locus of attention (see below), then we should obtain the same null result. Posner’s null finding could instead, however, have been due to a decision not to shift attention to the cued location because the subject believes that auditory stimuli have their own attention grabbing properties (Posner, Nissen and Klein, 1976) [23] which would obviate the need to orient attention. In that case, we would expect to obtain a cuing effect because orienting in response to peripheral visual cues is reflexive and not easily influenced by subject strategies (Jonides, 1981) [24].

#### 2.5.1. Methods

Six subjects were tested in 2 sessions each. Subjects received an uninformative visual cue that consisted of the illumination of the left or right LED for 250 ms or on neutral trials, the central LED was turned off for 250 ms. On non-catch trials, following one of three SOAS (0, 250 and 500 ms, with SOA order varied randomly across subjects) a 800 HZ tone was sounded on the left or right speaker for 250 ms. The subject’s task was to press the microswitch as soon as the tone target was detected. As in the previous experiments using a detection task, there was no target on 1/3rd of the trials.

#### 2.5.2. Results and Discussion

The results are shown in Table 5. Although there was a significant effect of cue condition (F (2, 10) = 9.93, *p* < 0.005), this is due entirely to the fact that the neutral cue condition was slower than both the cued and uncued conditions, which clearly do not differ. This effect is likely due to physical differences between the neutral and non-neutral cues in this experiment (onset of peripheral LEDs vs. brief offset of central LED). The effect of SOA was significant, with RT fastest in the 250 ms SOA condition (F (2, 10) = 6.11, *p* < 0.025).

We believe, with Posner (1978) [1], that the absence of any cuing effect is attributable to properties of the target modality—audition—rather than to properties of the cue modality—vision, or to a visual/auditory interaction. It has previously been demonstrated (Klein, 1977 [15]; Posner, Nissen and Posner, Nissen and Klein, 1976 [23]) that auditory stimuli are less influenced by the prior direction of attention because they have a capability similar to the “interrupt” in a computer which instantly summons the central processor to deal with certain stimuli. Under this view, the lack of a cuing effect with auditory stimuli is attributed to the relative insensitivity of auditory stimuli to the spatial locus of attention. Alternatively, visual and auditory events may produce orienting within visual space, but perhaps neither produces orienting within auditory space. Buchtel and Butter (1988) [8] make such a proposal, arguing that in vision there is a linkage between the mechanisms for overt and covert orienting [25], and that in contrast to the eye movement system, “there are no orienting movements of the auditory system that serve to improve identification of auditory stimuli” (Buchtel and Butter, 1988, [8] (p. 508). Further experimentation will be necessary to distinguish between these and other possible explanations of this cross-modal orienting asymmetry. 

### 2.6. Conclusions

We have demonstrated: (1) that following an uninformative peripherally presented tone subjects respond more rapidly to visual stimuli in the same location as the tone, as if visual attention had been exogenously drawn to that location; (2) that this shift of attention can last at least 500 ms and at least at the 250 ms SOA is not likely due to a lowered criterion for responding to information accruing from the stimulated location; (3) that it can be overcome via endogenous control of attention within 500 ms and reversed within 1000 ms when the tone indicates that the visual event is likely to be presented elsewhere, (4) that pitch glides and relative pitch do not elicit shifts of visual attention along the vertical meridian, and (5) that uninformative peripheral visual stimulation does not affect the speed of auditory detection.

Posner, Nissen and Ogden (1978) [26] produced evidence showing that endogenous orienting to a location in space is quite useless (or not possible) if the modality of the target is unknown. They inferred that the representation of space in which attention operates is not the multi-modal “common sense” that is so apparent in our phenomenological experience. Instead, attention operates best within the spatial map of an individual sensory modality. Our finding that visual detection can be hastened by a simultaneous or preceding tone at the same spatial location seems to support a multi-modal view of attention and space. We suggest that the distinction between 2 attentional mechanisms underlying endogenous and exogenous orienting (see introduction) may be the key to understanding the discrepancy. Attention that is pulled exogenously by localizable stimulation may operate in a multimodal representation of space. Such exogenous shifts may also be intimately linked to overt orienting mechanism (see also Buchtel and Butter, 1988) [8]. On the other hand, attentional shifts produced endogenously by the subject in response to probabilistic information, may involve the generation of an expectancy that affects processing within a sensory modality.

## 3. Part II: Frequency Glides and Endogenous Cross-Modal Orienting of Attention

In Experiment 1 of Part I we found that neither frequency glides nor high and low tones presented from straight ahead did not generate visual orienting along the vertical meridian. We cannot conclude from these negative findings with pitch and pitch change that exogenous visual orienting in response to such stimuli never occurs. One possibility that might be worth exploring is that a 21° shift of visual attention is too large for the pitch domain to elicit. In the present study we moved the visual targets (LEDs) closer together to explore this possibility. Another possibility (and one that is consistent with the artistic use of pitch to indicate direction) is that the pitch dimension does not produce exogenous orienting, but does activate a more cognitive or semantic level of spatial representation (see Marks, 1987) [27]. This view will be tested by making the stimuli in the pitch domain informative about the likely location of a visual event. If the “semantic” interpretation is correct, we would expect to see more rapid endogenous orienting when the probability information is consistent with the “meaning” naturally conveyed by the pitch properties of the auditory stimuli, and conversely, orienting should be considerably slowed when the two “meanings” are placed in conflict.

### 3.1. Experiment 6

This experiment is a close replication of the frequency glide condition of Experiment 1 with eye monitoring and a substantial reduction in the eccentricity of the visual targets. Importantly the direction of the frequency glide was, as in Experiment 1, completely uninformative about the location of the upcoming visual target.

#### 3.1.1. Methods

**Figure 3:**
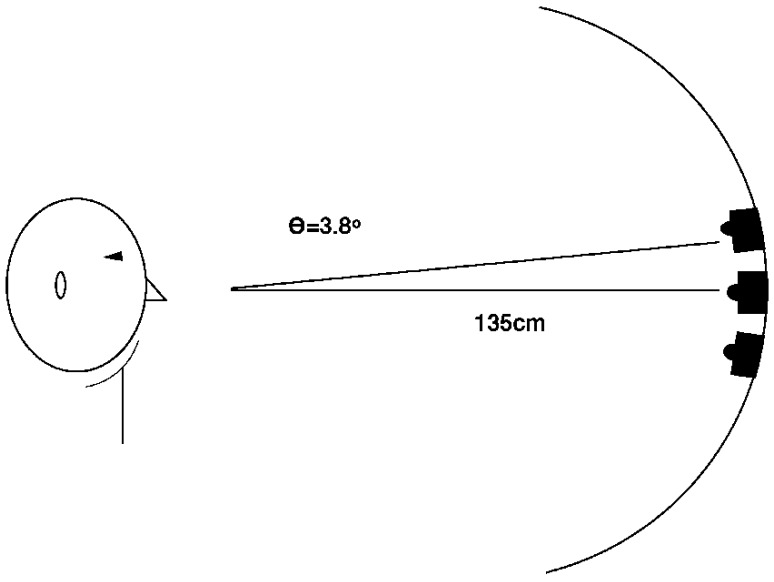
Relative position of apparatus and subject for Experiments 6 and 7.

Twelve subjects were tested. Those taking classes at Dalhousie University received credit points for participation. The remaining subjects volunteered without having been paid. The mean age was 27 years and 10 subjects were female. The methods were the same as the vertical orientation condition of Experiment 1, with the following exceptions. The peripheral speakers (not used) and LEDs were moved closer together as illustrated in Figure 3 such that there was a 3.8° visual angle between the central LED and the peripheral LEDs. Before the first test block of each SOA there was an 18 trial practice block. Three test blocks for each SOA were given and each comprised 72 randomly ordered trials. Eye position was monitored as in Experiments 2–5 and all trials with eye movements were replaced. Each test block had 16 trials with the target occurring at the corresponding location (e.g., rising pitch with top LED), 16 trials with the target occurring at the non-corresponding location, 16 neutral trials (steady tone and top or bottom LED) and 24 catch trials (no target following the auditory cue; 8 neutral tone cues, 16 non-neutral tone cues). The relationship between auditory stimulus and location of visual cue was completely random. Subjects were carefully made aware of this.

#### 3.1.2. Results

**Figure 4:**
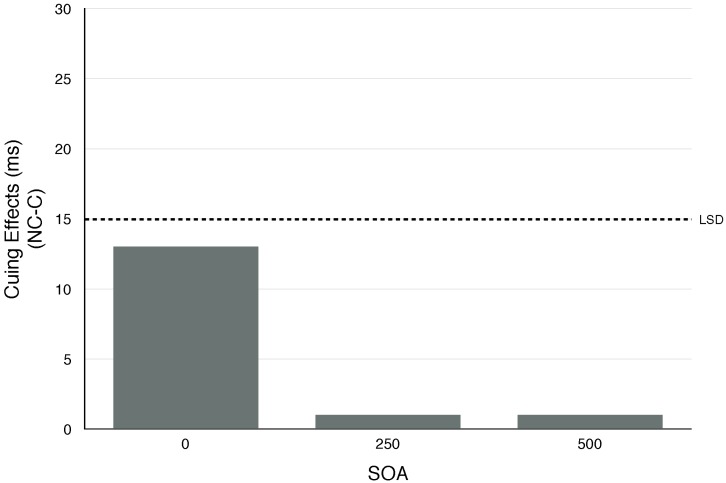
Cuing effects (NonCorresponding [NC] minus Corresponding [C] for Experiment 6. (LSD = Fischer’s least significant difference; values exceeding LSD are significant).

The results are shown in Table 6. Statistical analysis showed significant effects of SOA and cue condition (F (2, 22) = 3.80, *p* < 0.05 and F (2, 22) = 4.38, *p* < 0.01, respectively). However the significant effect of cue condition is due to slower reaction times for the neutral condition. Fischer’s least significant difference tests (LSD = 16.63) revealed that the cuing effects at all three SOAs were not significant. In other words, no exogenous covert orienting was produced within the 500 ms period tested (see Figure 4). These results replicate what we found in Experiment 1 with a much larger eccentricity of the visual targets.

### 3.2. Experiment 7

In this experiment the rising and falling pitch contours were made informative. In one condition the probability manipulation was compatible with the conventional meaning of rising and falling pitch contours (signaling upper and lower visual targets, respectively). In the other condition, the probability manipulation was incompatible with conventional meaning. In addition, we dropped the 250 ms CTOA and replaced it by a longer one (990 ms) in case it might take this long for participants to endogenously shift visual attention in response to the probabilistic information conveyed by the frequency glides.

#### 3.2.1. Methods

Twenty subjects, ten in each condition, participated in the experiment. The one subject who was taking classes at Dalhousie University received credit points for his participation; the remaining subjects were all volunteers and were not paid for their participation. The mean age was 28 years; 13 of the subjects were female. The procedure used in this experiment differed from that used in experiment one in some important respects. Subjects were randomly assigned to one of two conditions: Compatible or Incompatible. For the Compatible group 75% of non-catch trials (those with a target) exhibited a correspondence between the pitch glide and the location of the illuminated LED. In other words, for most trials subjects were presented with a rising pitch glide and the top LED, or a falling pitch glide and the bottom LED. The Incompatible group experienced a predominantly (75%) reversed, or non-congruent relationship between pitch glide and LED location. Subjects were carefully instructed on the relationship between the auditory cues and visual targets appropriate to their condition. Subjects were tested in one session using three SOAs (0 = simultaneous, 500 and 990 ms). The six order combinations were used once for the first 6 subjects in a group; for the remaining four subjects one of the 6 orders was randomly selected without replacement. A 20 trial practice block preceded the first test block of each SOA. There were two test blocks for each SOA. Although each test block contained 80 randomly ordered trials, because trials in which eye movements were made were replaced subjects might have experienced more than 80 trials. Each test block had 48 trials that supported the “rule” the subjects had been given (i.e., for the Compatible group: rising-top and falling-bottom, and for the Incompatible group: rising-bottom and falling-top), 16 trials inconsistent with the rule and 16 catch trials.

#### 3.2.2. Results

**Figure 5:**
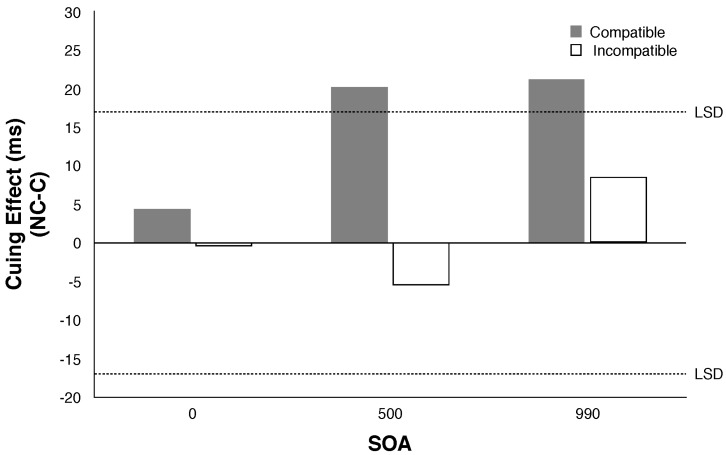
Cuing effects (NonCorresponding [NC] minus Corresponding [C] for Compatible and Incompatible groups in Experiment 7. (LSD = Fischer’s least significant difference; values exceeding LSD are significant). Note that “correspondence” refers to correspondence between the natural meaning of the pitch contour and the location of the target. If participants in the Incompatible group were using the probabilistic information conveyed by the cues’ pitch contour their scores would be significantly negative in this figure.

The results are shown in Figure 5. Using Fischer’s least significant difference, significant cuing effects were found for the Compatible group at SOAs of 500 and 990 ms (LSD = 16). The Incompatible group showed no significant cuing effects in either the direction of the “rule” (non-congruent) or in the direction of “natural meaning” (congruent) (see Figure 3).

### 3.3. Conclusions

The results from Experiment 6 demonstrate, in agreement with those of Experiment 1, that a rising or falling pitch contour does not exogenously (or reflexively) orient visual attention toward locations above or below the horizontal meridian respectively. The results from Experiment 7, however, demonstrate an important role for the natural meanings we attach to rising and falling pitch contours. In the Compatible condition subjects were able to endogenously shift their visual attention in the congruent direction and they could do this within 500 ms. In the Incompatible condition, however, incongruent shifts were not observed even at 990 ms. It appears, therefore, that endogenous cross-modal attentional shifts in response to pitch contours are possible, but only when a phenomenal correspondence (i.e., compatibility) exists between pitch glide and location. That such cross-modal shifts seem to require the mediation of “natural meaning” between visual and auditory information poses an interesting puzzle: With regard to the control of visual attention by auditory stimuli, the natural meaning of some stimuli is difficult to overcome endogenously even though such stimuli do not elicit exogenous control of visual attention.

## 4. Part III: Putting Old Findings about Covert Cross-Modality Orienting into a Contemporary Context

My principal goal in this final section of the paper is to explore the degree to which the findings reported in the previous sections have been confirmed, and if they haven’t been confirmed to explore the possible cause(s) of the discrepancy. The field of multi-sensory integration has exploded since my earliest work on this topic (conducted during my graduate career at the University of Oregon, and presented in these publications: Klein, 1977 [15]; Klein and Posner, 1974 [28]; Posner, Nissen and Klein, 1976 [23]). Whereas in the late 1980s I was still quite well informed about the multi-sensory literature, it would be imprudent to suggest that this is true now. Therefore, the task of determining the conceptual implications of the “old” work presented here for contemporary ideas about multi-sensory processing should be left to today’s experts.

When considering the degree to which the findings about cross-modal orienting reported here have been replicated, it is important to remain focused on the two adjectives in the title of this paper: “Covert exogenous”. With that focus established, let’s examine each of the findings reported above.

### 4.1. Localizable Auditory Stimuli Generate an Exogenous Shift of Covert Visual Attention

The finding, from Experiments 1–4, that localizable auditory stimuli generate an exogenous shift of covert visual attention was confirmed in Experiment 1 of a very thorough paper on this topic by Spence and Driver (1997) [29]. As we did in Experiment 4, Spence and Driver used a target task that required a 2-AFC to ensure that their evidence for a covert shift of attention was not simply due to a criterion shift. In their comments on the work presented in Part I, of which they were aware, Spence and Driver noted that our evidence from Experiment 4 might have been compromised by a speed-accuracy tradeoff because accuracy on valid trials was slightly worse than in the other conditions (see Table 4). Whereas it was probably appropriate for them to point this out (to enhance the empirical value of their findings) there are two reasons why our RT evidence for covert cross-modality orienting in Experiment 4 is unlikely to be compromised by a criterion shift. First, the RT effect was highly significant and the accuracy effect was not significant (F < 1). Secondly, Spence and Driver essentially replicated our finding. Hence, while a criterion shift explanation cannot be ruled out for the findings from Experiments 1–3, it is likely (and more parsimonious to assume) that the speed of processing (detecting and discriminating the properties of) visual targets was affected by their spatial position relative to the auditory cue. Indeed, Lee and Spence (2017) [30] began a recent exploration of the spatial precision of such cuing effects by describing this finding as: “One of the most oft-replicated findings in the field of exogenous crossmodal spatial attention research …”.

In all of the studies of which I am aware, that have explored visual orienting toward the spatial positions of uninformative auditory cues, the sources of the auditory cues were visible and the visual targets were presented at the same locations where the auditory stimuli could be presented. The question that was posed in most of these experiments was “will visuo-spatial attention be automatically captured by a localized auditory stimulus”. It is reasonable to ask whether such auditory cues would be equally effective in capturing visuo-spatial attention if their sources were not visible and if their were a sufficient number and distribution of them to disrupt the participant’s ability to confidently link them with the spatial positions of the visual targets.

### 4.2. Such Cross-Modality Cuing Effects Are Relatively Automatic

The term “relatively” is intended, here, to anticipate several reactions. Whereas, due to my training I have been strongly influenced by the criteria for automaticity that were put forward by Posner and Snyder (1975) [31], I recognize that there is considerable disagreement on this topic. Secondly, the research reported here only explores one criterion: “that the effect takes place regardless of our intentions”. Finally, “strong” automaticity implies not only that effects take place despite our intentions but also that these effects will not be modified by our intentions, an immunity that seems highly unlikely in studies of exogenous covert orienting (e.g., see Folk, Remington and Johnston, 1992) [32].

The use of uninformative spatial cues in the Posner cuing paradigm is intended to give the observer no incentive to attend in the direction of cue. It is generally assumed that when cuing effects are observed they are due to involuntary capture of attention by the cue. In Experiment 3 we gave our participants an incentive to use the auditory cues to direct their visual attention toward the uncued (opposite) location. As illustrated in Figure 2, when so incentivized to attend away from the auditory cues the cuing effect at the shortest SOA we tested (0 ms) was at least as large, if not larger, than when the cues were simply uninformative. This automatic capture of attention by the cue could be reversed by endogenous control, but this required over half a second [33]. 

Finally, it should be noted that inhibition of return, which has been demonstrated to operate cross-modally (Spence et al., 2000) [34], might be contributing to the negative cuing effect at the 1 s CTOA. It is unlikely to be contributing much at 500 ms because at this interval cuing was still positive when the auditory stimulus was uninformative.

### 4.3. Localizable Visual Stimuli Do Not Generate an Exogenous Shift of Covert Auditory Attention

The effect of uninformative but localizable visual stimuli on attention in the auditory modality was also explored in the aforementioned study by Spence and Driver (1997) [29]. Their findings are exceptionally informative. In two experiments with both visual and auditory targets, the visual cues, whether onsets (Experiment 3) or offsets (Experiment 4), generated robust cuing effects when the targets were visual and no cuing effects when the targets were auditory. This confirms what is reported in Experiment 5 and extends this observation in two ways: firstly from a detection to a discrimination task, and secondly by including visual targets. It is remotely possible that in our Experiment 5 participants adopted a modality-based attentional control setting in which the visual cues were ignored because with no visual targets there was no need to attend vision at any point in the trial. In their Experiment 5, however, which eliminated the visual targets, Spence and Driver found a small but significant cuing effect so long as the SOA was greater than 100 ms (cuing effects were 2, 8 and 9 ms at SOAs of 100, 200 and 700 ms). Eye position was not monitored in this experiment and to test the possibility that overt orienting might have contributed to this pattern of results in their final experiment two groups of participants were tested: one with and one without eye monitoring. The results were striking: “there was no overall validity effect when eye movements were monitored (mean invalid minus valid difference of 0 ms), but a mean valid advantage of 7 ms for unmonitored subjects”. Because in our Experiment 5 eye position was monitored assiduously and participants were warned when untoward movements were detected, I completely agree with Spence and Driver’s conclusion: “… uninformative visual cues do not result in exogenous shifts of auditory attention, with the added caveat that this applies only under conditions in which overt orienting is prevented”.

### 4.4. Neither the Frequency Nor the Direction of a Frequency Glide Generates an Exogenous Shift of Covert Visual Attention

The inability of uninformative frequency glides to generate exogenous shifts of visual attention was demonstrated in the vertical condition of Experiment 1 and also in Experiment 6. In addition, as reported in [13], a similar absence of visual cuing effects was observed when the auditory cues were the relative frequency or pitch of unvarying tones. As discussed in a recent review (Spence and Deroy, 2013) [35], these null results are anomalous. Focusing on those studies designed to determine whether pitch or pitch glides might direct exogenous attention along the vertical axis, there are three papers (see Table 1 in Spence and Deroy) that reported significant cuing effects from pitch and/or pitch glides: Chiou and Rich (2012) [36], Fernández-Prieto, Vera- Constán, García-Morera, and Navarra (2012) [37]; later published on line, Fernández-Prieto and Navarra, 2017 [38]) and Mossbridge, Grabowecky, and Suzuki (2011) [39]. These are all fine papers that employed interesting and revealing manipulations. Here we will examine pitch and pitch glides separately.

**Figure 6:**
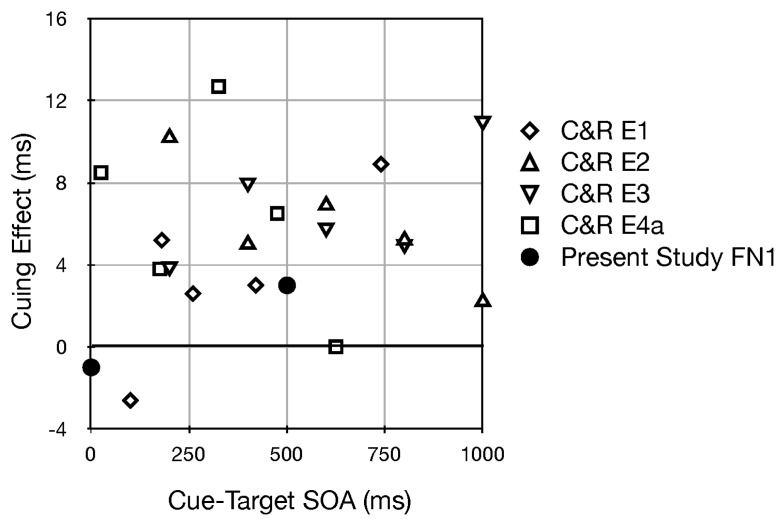
Visual cuing effects along the vertical meridian generated by steady state auditory stimuli differing in pitch. Positive scores indicate that high/low tones resulted in faster reaction times for higher/lower visual targets, respectively. The experiments from Chiou and Rich (2012) [36] are indicated by C&R.

The only finding reported here that was about the possibility of visual orienting in the vertical dimension in response to static pitch (high versus low frequency tones) was briefly described in [13]. Those findings are presented in Figure 6 along with the results from 4 experiments in Chiou and Rich (2012) [36] that used uninformative cues. Several points are worth noting. The average cuing effect, which was significant in each of the 4 experiments from Chiou and Rich, is rather small, at 5.6 ms. Even though our finding of a 3 ms cuing effect at an SOA of 500 is smaller when plotted against the somewhat noisy data points from Chiou and Rich it does not look anomalous. Finally, the number of participants we tested was 6 whereas in Chiou and Rich’s experiments the number of participants ranged from 12 to 18. Based on these points, I think it is reasonable to conclude that uninformative pitch weakly generates covert visual orienting exogenously. Two qualifications based on the findings from Chiou and Rich’s study are worth noting: In their Experiment 2 they did not obtain any cuing effect when the pitch difference was small (300 vs. 400 Hz) and in Experiment 3 they demonstrated that it is relative pitch in the context of the experiment that matters, not absolute pitch of the auditory cues.

**Figure 7:**
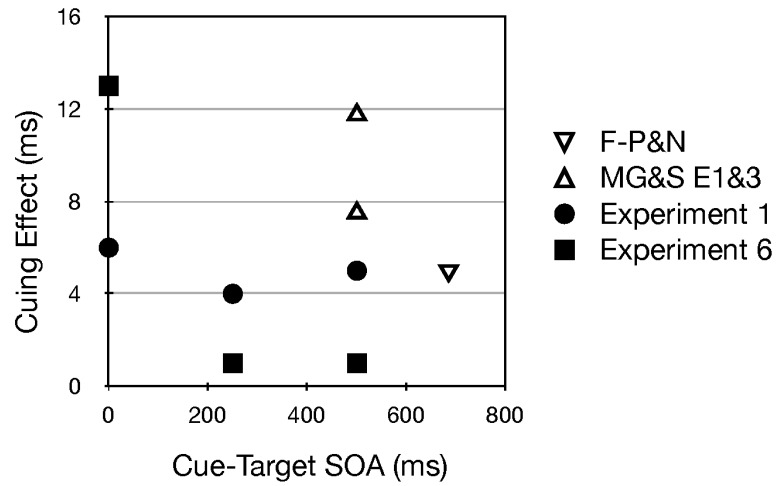
Visual cuing effects along the vertical meridian generated by auditory glides. Positive scores indicate that rising/falling tones resulted in faster reaction times for higher/lower visual targets, respectively. The experiment from Fernández-Prieto and Navarra (2017) [38] is indicated by F-P&N and the experiments by Mossbridge et al. (2011) [39]. SOA is timed from the beginning of the frequency glide.

In Experiments 1 and 6 uninformative frequency glides did not significantly affect visual detection latencies along the vertical axis. Statistically speaking, this finding conflicts with what was reported by Mossbridge et al. (2011) [39] and Fernández-Prieto and Navarra (2017) [38] (see Figure 7). Because it seems likely that a 0 ms SOA is too short to permit orienting in response to a frequency glide that is beginning at time 0, and because these other studies only used longer SOAs, I believe it is reasonable, when exploring the source of the empirical conflict, to concentrate on the longer SOAs. Each of these studies has a feature that causes me to be cautious about putting too much weight on their findings.

Mossbridge et al. [39] used an interesting go/nogo matching to sample task. At the start of a trial, and during the frequency glide, a colored disk was presented at fixation for 500 ms. Then, as indicted in the methods: “Upon offset of the reference circle and sound, the probe circle appeared in one of the four squares for 750 ms”. Given the relatively low salience of the remaining fixation dot, the lack of eye monitoring, the peripheral color discrimination required for correct responding and the fact that all RTs in this task came from trials for which the colors of the disk at fixation and the immediately following probe disk in the periphery matched, this method was almost certain to elicit saccadic eye movements. Consequently, the orienting explored in this otherwise excellent study was unlikely to have been covert. Fernández-Prieto and Navarra (2017) [38] used a simple detection task as we did (in all but Experiment 4). The use of catch trials in simple detection tasks is considered de rigueur among reaction time experts. However, unlike our methods, which included a relatively high percentage of catch trials (33%), no catch trials were used by Fernández-Prieto and Navarra. Consequently, I am weakly inclined to believe that auditory frequency glides do not orient covert visual attention exogenously. Putting aside these concerns and ignoring the rational for focusing on the longer SOAs, it must be noted that the average cuing effect in our two studies (all filled symbols in Figure 7) is about 5 ms. Because this is very similar to the small but significant effect of static pitch (Figure 6), a cautious approach would be to recommend further data collection [14].

### 4.5. The Endogenous Use of Pitch Glides Is Influenced by Their Natural Meanings

When, in Experiment 7, we made the rising and falling pitch contours informative about where the visual targets would appear, we found that our participants were able to use these stimuli to attend in the directions compatible with their natural meaning (rising-up; falling-down); but they were not able to do so when probability manipulation was incompatible their natural meaning. To our knowledge, there is no other published study that has explored this possibility, so we are inclined to take it at face value. There is, however, one study (Chiou and Rich (2012) [36], Experiment 4b) that employed the incompatible manipulation with highly (counter-)informative (80:20) static pitches (high tones signaled visual targets would appear below fixation; low tones signaled they would appear above). In this condition, at SOAs of 350 and 650 ms their participants showed a 21 ms advantage for visual targets at the expected location. Although it is certainly possible that both results are correct, this is another question for which further data collection would be useful.

## 5. Epilogue

I would like to end with a few comments and recommendations for future research. When considering empirical conflicts in their wide-ranging review of cross-modal correspondence effects, Spence and Deroy (2013) [35] noted that: “there could be subtle differences attributable to the particular stimuli (and, more importantly, the range of stimuli) that researchers happen to have used (see Table 1) which bear on the salience of the relevant dimensions”. I completely agree and would like to add that researchers should also consider whether the reflexive oculomotor system of their participants is suppressed by eye monitoring and feedback about untoward eye movements or not so suppressed. Importantly, this distinction is not simply about separating trials with from those without eye movements; it is about whether the conditions of the experiment do or do not encourage suppression of reflexive saccades. Recent work on inhibition of return in my lab (for a review see, Klein and Redden, in press) [40] suggests that entirely different forms of IOR are generated depending on the state of the reflexive oculomotor system. It is possible that the same might be true for the nature of cross-modal correspondence effects upon covert orienting. Although from theoretical and methodological points of view this distinction is of considerable importance, because an unsuppressed reflexive oculomotor system is a regular feature of everyday experience, from a practical point of view, we might be more interested in those cross-modal correspondences associated with that mental state.

As for the empirical generalizations that were obtained in our experiments from the 1980s, our findings that localizable auditory stimuli can covertly and exogenously capture visual attention while localizable visual stimuli do not similarly capture auditory attention are well reinforced. Our finding that pitch did not significantly drive attention along the vertical axis has been challenged. Because when positive effects are observed they are very small, and because our power to detect small effects was low, I am open to the possibility that our conclusion of null effects on this topic was incorrect. Our similar null finding with pitch glides has also been challenged, but methodological weaknesses in the challenging studies allow for the possibility that our null finding is true. With regard to the degree to which pitch glides might be used to endogenously and covertly guide visual attention to the location where a visual target is likely to appear I am confident that when the probability manipulation is compatible with the normal meaning assigned to such glides, participants can effectively use this information. A confident conclusion for the incompatible mapping will require further investigation.

Finally, I regularly advise my students (cf Tversky and Kahneman, 1971) [41] that for our discipline the cost of a Type 2 error can be greater than that of a Type I error. Suppose one proposes an interesting hypothesis and conducts a study that, in its favor, rejects the null. If your hypothesis were really wrong and the null is true, the field will eventually figure this out (though various publication biases may have to be overcome, e.g., see Klein, 2015 [42]) because interesting hypotheses are likely to be subject to follow-up research. Some researchers might find this embarrassing but we can and should get over it. On the other hand, suppose you have a interesting hypothesis that is really true, but you conduct an experiment with a null result and, making a Type II error, you conclude it to be false. Rather than try to publish your null finding you put the project in a file drawer. In this case, it is possible that your interesting, and maybe even important, idea will never be rediscovered. The message I should have been more sensitive to back in the 1980s (and which today—fortunately—seems to have become conventional practice) is “do not accept the null when your study is underpowered” and conversely, “when you have a good idea invest sufficient power in your test of it”.

## Figures and Tables

**Table 1:** Reaction times and cuing effects (cued minus uncued) for the left/right (top panel) and rising/falling (bottom panel) of Experiment 1.

	SOA		
Left/Right	0	250	500
Cued	305	288	303
Neutral	319	306	316
Uncued	322	314	317
Cuing Effect	17	26	14
Rising/Falling	0	250	500
Valid	325	316	319
Neutral	331	320	325
Uncued	331	320	324
Cuing Effect	6	4	5

**Table 2:** Reaction time and cuing effects from Experiment 2 (left/right condition with monitoring of eye position).

		SOA	
	0	250	500
Valid	331	311	336
Neutral	335	323	342
Uncued	337	334	351
Cuing Effect	6	23	15

**Table 3:** Reaction time and cuing effects from Experiment 3a (top panel) and Experiment 3b (lower panel) in which the visual targets were likely to appear at the location opposite to that of a left/right tone. Eye position was monitored. Note: cued and uncued refer to the locations of the auditory tones, and not to the locations likely to contain the target (which, given this terminology, are uncued and cued, respectively).

	SOA		
Experiment 3a	0	250	500
Cued	330	342	366
Neutral	350	354	372
Uncued	350	346	361
Cuing Effect	20	4	−5
Experiment 3b	0	500	1000
Cued	330	373	391
Neutral	351	373	369
Uncued	351	353	369
Cuing Effect	21	−10	−22

**Table 4:** RT and errors from Experiment 4 in which subjects made a 2-choice discrimination to a visual target that appeared 250 ms after the onset of a left/right tone.

	RT	% Error
Cued	612	20.0
Neutral	629	19.3
Uncued	651	18.0

**Table 5:** Reaction time and cuing effects from Experiment 5 with visual cues and auditory targets.

		SOA	
	0	250	500
Cued	321	284	294
Neutral	326	304	319
Uncued	316	276	303
Cuing Effect	−5	−8	9

**Table 6:** Reaction times and cuing effects (NonCorresponding minus Corresponding). for Experiment 6 with top/bottom visual targets and rising/falling auditory cues.

	SOA		
	0	250	500
Corresponding	356	330	364
Neutral	362	345	375
Non-Corresponding	369	331	365
Cuing Effect	13	1	1
